# 

**Published:** 1895-04

**Authors:** 


### EDITORIAL.

The American Medical Association meets in Baltimore on May 7th prox. It will doubtless be a large and interesting meeting, at least the history of this body fully warrants this statement.

It is of interest to dentists to know that one of the important sections of this association is that of Oral Surgery. This section ought to elicit the attention and co-operation of the best men in the dental profession. Its members are connected with the leading medical associations of this continent and an opportunity is here afforded of hearing the principal topics of medical science and practice considered in papers and by discussion. It is gratifying to meet and know the men who have been instrumental in developing medical science. The section on oral surgery has already accomplished great good. The work done by it is said, by the medical members of the association, to be equal in character to that of other sections of the body. At every annual meeting members of other specialties, and general practitioners as well, read papers and discuss subjects in the dental section, and members of the dental section are often called upon to read papers and discuss subjects before other sections.

The American Medical Association is the great controlling power to prevent specialism from running wild, and yet fostering it in such a way as to develop the greatest amount of good.

Here specialties are brought together in such a way as to secure harmony and a realization of the fact that they are all but parts of one harmonious whole, and here we also learn that each of these may be helpful to the others. The fact is that all specialties in medicine have one common object, viz: the prevention and mitigation of human suffering and promoting the happiness of mankind.

It is very desirable that the specialty of oral surgery should avail itself of all the benefits derivable from an intimate connection with the American Medical Association.

### A Return.

We are gratified to learn that Dr. A. W. Harlan has returned to his "*first love.*" In other words, has again taken editorial charge of "The Dental Review." This will possibly be a surprise, but doubtless a gratifying one to the profession generally.

He was the first editor of the Review and no doubt feels at home on his return. The doctor can do his profession great service with his pen and he certainly should use it, as he wields the pen of a ready writer.

### Retirement.

We have just learned that Professor W. X. Sudduth has resigned as Dean and Professor of the College of Dentistry of the University of Minnesota, which will be a surprise to all his friends, and we doubt not a matter of regret on the part of the dental college.

He is, moreover, as we learn, to be retained as lecturer on special subjects.

This step has been taken by Dr. Sudduth in order that he may have time and opportunity to take up certain lines of advanced and original scientific work for which he had always a great desire. There are so few who have the ability and courage to enter upon the severe study and training involved in original scientific work, that it is gratifying to have one so well equipped for it, as is Dr. Sudduth, engage in it. He will enter upon this work, in the near future, in connection with the University of Chicago where large opportunities will be afforded him in his chosen field.

May his success be all that he and his friends can anticipate

### NOTICES.

### Mississippi Valley Dental Association.

SEMI-CENTENNIAL MEETING, APRIL 17 AND 18—HALL E, ODD FELLOWS' TEMPLE, SEVENTH AND ELM, CINCINNATI—1895.

PROGRAMME.

1. President's Address—Dr. J. Taft, Cincinnati. Discussers: Drs. J. N. Crouse, Chicago; L. P. Bethel, Kent; Frank Sage, Cincinnati.

2. "English Tube Crowns for Bridge-work," Dr. J. R. Callahan, Cincinnati. Discussers: Drs. Wm. V. B. Ames, Chicago; L. E. Custer, Dayton; A. F. Emminger, Columbus.

3. "History of the Society," Dr. E. G. Betty, Cincinnati. Discussers: Drs. Wm. H. Morgan, Nashville; C. R. Butler, Cleveland; H. A. Smith, J. Taft, Cincinnati.

4. "Rigg's Disease; or, 'What You Will,'" Dr. J. E. Cravens, Indianapolis. Discussers: Drs. A. W. Harlan, Chicago; J. S. Cassidy, Covington; O. N. Heise, Frank A. Hunter, Cincinnati.

5. "Crown-work," Dr. George Evans, New York City. Discussers: Drs. Grant Molyneaux, Cincinnati; C. I. Keely, Hamilton; Otto Arnold, Columbus.

6. "What a Dentist Saw in Examining Five Hundred Crania," Dr. M. H. Fletcher, Cincinnati. Discussers: Drs. J. E. Cravens, Indianapolis; C. M. Wright, E. G. Betty, Cincinnati.

7. "Tumors of the Mouth," Dr. C. G. Darling, Ann Arbor. Discussers: Drs. Truman Brophy, Chicago; M. H. Fletcher, Cincinnati.

Demonstrations—Electric Oven and Appliances for Accurately Determining the Heat—Dr. L. E. Custer, Dayton.

Practical Showing of Plastic Rubber, Etc.—Dr. A. S. Billings, Omaha.

### TOPICS FOR DISCUSSION.

(As suggested by the American Dental Association.)

No. 1. Can alveolar-dental abscess arise after complete sterilization and obliteration of the canal by an impervious filling; and if so, from what causes?

No. 2. What are the best means of diagnosis of pulp-calcification in its several forms? To what extent does the process demand treatment, and how shall it be treated, (*a*) with respect to its prevention, and (*b*) remedially?

No. 3. What is the most satisfactory antiseptic and best method for root-canal sterilization?

No. 4. Is not operative dentistry liable to the same injury from the too prevalent use of plastic stoppings as occurred to prosthetic practice from the introduction of vulcanite?

### LIST OF OFFICERS.

President—Dr. J. Taft, Cincinnati.

First Vice-President—Dr. Wm. V. B. Ames, Chicago.

Second Vice-President—Dr. A. F. Emminger, Columbus.

Treasurer—Dr. F. A. Hunter, Cincinnati.

Corresponding Secretary—Dr. H. C. Matlack, Cincinnati.

Recording Secretary—Dr. H. T. Smith, Cincinnati.

Executive Committee—Drs. J. R. Callahan, M. H. Fletcher, L. E. Custer.

### Notice.

### AMERICAN MEDICAL ASSOCIATION—DENTAL SECTION.

The forty-sixth annual session of the American Medical Association will be held in Baltimore, Md., on Tuesday, Wednesday, Thursday and Friday, May 7, 8, 9 and 10, commencing on Tuesday, at 10 A. M.

### SECTION ON DENTAL AND ORAL SURGERY.

Dr. M. E. Fletcher, Chairman; Dr. Eugene Talbot, Secretary.

Chairman's Address.

"Bacteriology," by S. A. Hopkins, Boston.

"Adenoids," by Geo. F. Eames, Boston.

"The Effect of Sterilizing Processes upon Steel Instruments," by Wm. H. Potter, Boston.

"Common Ground of Medicine and Dentistry," Joseph Roach, Baltimore.

"Destruction of Children's Teeth, Cause and Remedy," by J. G. Humsler, Baltimore.

"The Progress of Modern Dental Practice," by A. H. Thompson, Topeka, Kan.

"Suppuration of the Intermaxillary Bones with Fistulous Opening into the Nasal Cavity," two cases, by W. Xavier Sudduth, Minnesota.

"Ulceration of the Oral Cavity and Its Treatment," by Dr. Genese, Baltimore.

"Specific Treatment of Necrosis of the Alveoli and Maxillæ with Sulphuric Acid," by W. A. Mills, Baltimore.

"Diseases of the Soft Parts of the Mouth and Ill-Developed Jaws," by W. S. Twilley, Baltimore.

"A Presentation of Inflammation and Tumorous Growths Caused by Wearing Rubber Denture," by Bernard Myer, Baltimore.

"Calcification of the Teeth," by R. R. Andrews, Cambridge.

"The Value of Differential Diagnosis in Dentistry," by Vida A. Latham, Chicago.

"The Discovery of Anæsthesia and its Outgrowth, Rapid Breathing as a Pain Obtruder in Minor Surgery and Medicine, with its History and Application for Twenty Years," by W. Bonwill, Philadelphia.

### THE DENTAL REGISTER,

A MONTHLY MAGAZINE DEVOTED TO THE INTERESTS OF THE

**DENTAL PROFESSION.**

*Terms Reduced to* $2.00 *Per Year. Single Copies,* 25 *Cents.*

**EDITED BY PROF. J. TAFT, D.D.S.**

—AND—

**Published by SAM'L A. CROCKER & CO., Ohio Dental and Surgical Depot.**

The volume commences in January and closes in December.

The REGISTER being issued monthly is always fresh, therefore Indispensable to the practitioner who desires to keep posted up with the new inventions and improvements which are daily developed. Neither expense nor effort will be spared to give value and variety to its contents. Articles will be illustrated with well executed engravings whenever they will add to the interest or assist to explanation.

Its extensive circulation and frequency of issue make it one of the BEST DENTAL ADVERTISING MEDIUMS in the United States.

All subscriptions must begin with January or July.

Local or special notices, five lines or less, will be inserted for $3 each insertion; over five lines, 50 cts. per line.

All advertising bills payable in advance, or at furthest, after the issue of the first number containing the advertisement. All transient advertisements to be paid for in advance.

☞CONTRIBUTIONS SOLICITED.

Exclusively Editorial Communications should be addressed to the Editor.

The latest number of the REGISTER may be ordered with or without other goods at any time, and all letters should be addressed to

